# *EVI1* controls proliferation in acute myeloid leukaemia through modulation of *miR-1-2*

**DOI:** 10.1038/sj.bjc.6605874

**Published:** 2010-09-14

**Authors:** M Gómez-Benito, A Conchillo, M A García, I Vázquez, M Maicas, C Vicente, I Cristobal, N Marcotegui, L García-Ortí, E Bandrés, M J Calasanz, M M Alonso, M D Odero

**Affiliations:** 1 Division of Oncology, Center for Applied Medical Research, University of Navarra, Navarra, Pamplona 31008, Spain; 2 Department of Genetics, University of Navarra, Navarra, Pamplona, Spain

**Keywords:** AML, *EVI1*, MiR-1-2, MiR-133a-1

## Abstract

**Bakground:**

The *EVI1*(ecotropic virus integration site 1) gene codes for a zinc-finger transcription factor, whose transcriptional activation leads to a particularly aggressive form of acute myeloid leukaemia (AML). Although, *EVI1* interactions with key proteins in hematopoiesis have been previously described, the precise role of this transcription factor in promoting leukaemic transformation is not completely understood. Recent works have identified specific microRNA (miRNA) signatures in different AML subgroups. However, there is no analysis of miRNAs profiles associated with *EVI1* overexpression in humans.

**Methods:**

We performed QT-RT–PCR to assess the expression of 250 miRNAs in cell lines with or without *EVI1* overexpression and in patient samples. We used ChIP assays to evaluated the possible binding of *EVI1* binding to the putative miRNA promoter. Proliferation of the different cell lines transfected with the anti- or pre-miRs was quantified by MTT.

**Results:**

Our data showed that *EVI1* expression was significantly correlated with the expression of *miR-1-2* and *miR-133-a-1* in established cell lines and in patient samples. ChIP assays confirmed that *EVI1* binds directly to the promoter of these two miRNAs. However, only *miR-1-2* was involved in abnormal proliferation in *EVI1* expressing cell lines.

**Conclusions:**

Our data showed that *EVI1* controls proliferation in AML through modulation of *miR-1-2*. This study contributes to further understand the transcriptional networks involving transcription factors and miRNAs in AML.

The human *EVI1* (ecotropic virus integration site 1) gene is located on 3q26.2, a region frequently rearranged in acute myeloid leukaemia (AML) (Wieser, 2007). Most patients with 3q26 rearrangements overexpress *EVI1*. However, its expression is also elevated in about 10% of AML patients with no 3q aberrations, as a result of so far unknown mechanisms (Wieser, 2007). Of importance, *EVI1* overexpression associates with poor prognosis and a shorter survival in AML (Nucifora *et al*, 2006).

MicroRNAs (miRNAs) are small non-coding RNAs that negatively regulate gene expression by repressing translation or inducing sequence-specific degradation of target mRNAs (Bartel, 2004). MicroRNAs play important roles in numerous cellular processes including hematopoiesis (Shivdasani, 2006; Lawrie, 2007; Fatica, 2006 no. 13). Over the past 3 years, several groups have described miRNA signatures associated with recurrent cytogenetic, molecular aberrations and with outcome in AML (Fabbri *et al*, 2008). However, anything is known about a miRNA profile in human AML patients with 3q26 rearrangements or *EVI1* overexpression. It has been reported that miRNA regulation is mediated by lineage-specific transcription factors involved in the developmental and differentiation processes (Bartel, 2004; Choong *et al*, 2007; Fazi *et al*, 2005). Interestingly, *EVI1* and miRNAs have important roles in development and cell differentiation (Perkins *et al*, 1991). In this study, we establish a miRNA signature in AML that could be possibly regulated by overexpression of *EVI1*. We found a strong positive correlation between the expression levels of *EVI1* and clustered microRNAs *miR-1-2/133a-1* in AML cell lines and in patient samples. We demonstrated that *EVI1* binds to a region upstream of the miRNA cluster resulting in an upregulation of *miR-1-2/133a-1*. Furthermore, functional studies showed that expression of *miR-1-2* was associated with enhanced proliferation in AML.

## Materials and methods

### Cell lines and patient samples

Cell lines MUTZ-3, TF-1, F-36P, HEL, HL-60, NOMO-1, MOLM-13, and OCI-AML2 were maintained in RPMI-1640, supplemented with 1% penicillin-streptomycin, and 10% FBS (GIBCO-BRL, Grand Island, NY, USA); 10 ng ml^–1^ GM-CSF was added in MUTZ-3, TF-1, and F-36P. P19 cell line was maintained in *α*MEM, supplemented wit 10% FBS. Patient samples were collected from the Department of Genetics, University of Navarra, Spain (Supplementary Table 1). Leukaemic blasts were obtained from bone marrow of AML patients with more than 60% blasts. The study was carried out in accordance with the ethical guidelines of our institution.

### Quantitative RT–PCR (qRT–PCR) of mRNA and miRNA levels

Quantitative RT–PCR was carried out with ABI Prism-7500 (Applied Biosystems, Foster City, CA, USA) with 20 ng of cDNA using TaqMan gene expression assays for *EVI1* (Hs01118675_m1) and human GAPDH (Hs99999905_m1). Overexpression of *EVI1* transcript was defined as levels higher than the average of seven bone marrow samples from healthy volunteers and three times the standard deviation. For miRNA quantification, qRT-PCR was performed with 10 ng of total RNA using either the TaqMan miRNA Human Panel Assay Set (ED000298) or TaqMan miRNA individual assays for miR-1-2 (002222), miR-133a-1 (002246), miR-146b (001097), miR-155 (002623), miR-323 (000538), miR-379 (000568) and snRNA U6B (Applied Biosystems).

### Western blot analysis

Nuclear or cytoplasmatic protein samples were resolved by SDS–PAGE, electroblotted to a nitrocellulose membrane (Bio-Rad, Hercules, CA, USA) and incubated with the appropriate antibodies: anti-*EVI1* (no. 2265, Cell Signaling), anti-lamA/C (no. 2032, Cell Signaling) and anti-*β*-tubulin (T4026, Sigma-Aldrich, St Louis, MO, USA).

### Chromatin immunoprecipitation assay

10 × 10^6^ HEL cells were used with ChIP assay kit (Upstate, Syracuse, NY, USA) according to the manufacturer instructions. Protein–DNA complexes were immunoprecipitated overnight with 5 *μ*g of anti-*EVI1* (C-20) mouse antibody (sc-8707-X, Santa Cruz Biotechnology, Santa Cruz, CA, USA). Genomic regions containing the *EVI1*-binding sites in the pre-miR-1-2 or pre-miR-133a-1 upstream regions (Ensembl 52 miRNA genes: ENSG00000207694 and ENSG00000207786) were amplified by PCR using specific primers flanking the *EVI1* binding sites: oligo no. 1 (forward), 5′-aaacccaggtgctcacagac-3′ oligo no. 1 (reverse), 5′-cattccatagcattgtatgttca-3′ oligo no. 2 (forward), 5′-ttggcaatctgtacccaaaa-3′ oligo no. 2 (reverse), 5′-tttcctgcgcttaatggttt-3′. Quantification of coimmunoprecipitated promoter fragments was performed in triplicate using the SYBR-Green dye detection with oligos no. 1.

#### All-trans retinoic acid (ATRA) and DMSO treatments

All-trans retinoic acid (ATRA) and DMSO (Sigma-Aldrich) were used as previously reported (Kazama *et al*, 1999). Cell morphology was evaluated in conventional light-field microscopy.

#### Transfection assays

P19 cells were transfected with siGENOME siRNA D-006530-06 (Dharmacon, Lafayette, CO, USA) by Lipofectamine 2000 (Invitrogen, Carlsbad, CA, USA) 48 h after the ATRA treatment; RNA was extracted 48 h after transfection. For miRNA functional assays, HEL or HL-60 cells were nucleofected (Amaxa technology, Gaithersburg, MD, USA) with pre-miR-1-2 precursor (PM10617), pre-miR-1-2 inhibitor (AM10617), pre-miR-133a-1 precursor (PM10413), pre-miR-133a-1 inhibitor (AM10413), pre-miRNA, or anti-miRNA scrambled control (Applied Biosystems).

### Statistical analysis

Class comparison and significant analysis of microarrays (SAMs) were performed to identify differentially expressed miRNAs. To make the analysis more restrictive and avoid false positives, *P*<0.01 was interpreted to denote statistical significance. To evaluate the correlation between *EVI1* and miRNAs levels expression in cell lines and patient samples we used Spearman's rank correlation coefficient because *EVI1* values lacked a normal distribution. ΔCt *EVI1* (Ct *EVI1*-Ct GADPH) and ΔCt miRNA (Ct miRNA-Ct snRNA U6B) were used instead of raw data Ct as a way to normalise data. A value of *P*<0.05 was interpreted to denote statistical significance. Statistical analysis was performed using the SPSS 15.0 statistical package.

## Results

**Figure 1:**
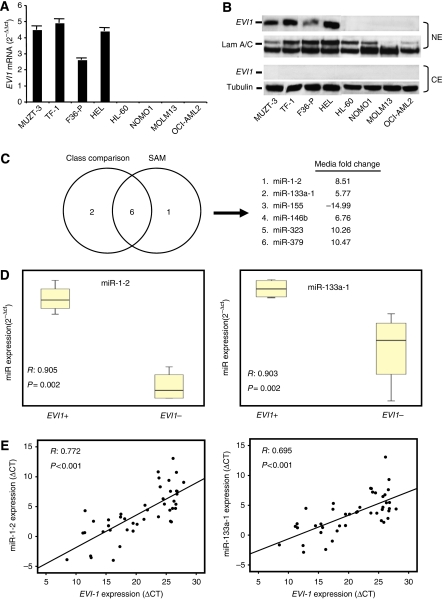
*EVI1* and miRNAs expression in AML. (**A**) Relative qRT–PCR quantification of *EVI1* transcript expression in AML cell lines. The GADPH levels were used as a normaliser for the calculation of the 2^−ΔΔCt^ coefficient. (**B**) Immunoblot analysis of *EVI1* protein levels in nuclear (NE) and cytosolic fractions (CE). Lam A/C and tubulin levels serve as control for equal protein loading. (**C**) Identification of miRNAs differentially expressed (*P*<0.01) between *EVI1*+ and *EVI1*– cell lines by class comparison and significant analysis of microarrays (SAM) methods. (**D**) Statistical analysis of *miR-1-2* and *miR-133a-1* expression levels in *EVI1*+ and *EVI1*− cell lines. (**E**) Spearman's rank correlation coefficient between ΔCt *EVI1* and ΔCt *miR-1-2* or *miR-133a-1* in patient samples.

To identify *EVI1*-miRNA signatures in AML, we choose eight cell lines previously reported to be either positive or negative for *EVI1* expression. We confirmed *EVI1* expression at the mRNA and protein level in these cell lines (Figure 1A and B) and we analysed the expression levels of 250 mature miRNAs by qRT–PCR. After raw data Ct normalisation, statistical analysis was performed by class comparison and SAM to identify differentially expressed miRNAs between the two groups. Several miRNAs were identified by using each method, and six of them were common to both lists (Figure 1C). Among these miRNAs, we found that miR-1-2 and miR-133a-1 showed the highest correlation coefficient with *EVI1* expression (*R*=0.905 and *R*=0.902, respectively) and the best significance value (*P*=0.002) (Figure 1D). Thus, we decided to validate these two candidates in a cohort of 44 AML patient samples (20 with *EVI1* overexpression). We found a significant correlation between the *EVI1* mRNA and both miRNAs expression levels (*R*=0.772 and 0.695 for miR-1-2 and miR-133a-1, respectively; *P*<0.001) (Figure 1E). Thus, *EVI1* may be involved in triggering or maintaining miR-1-2 and miR-133a-1 expression in AML and favouring the perpetuation of the neoplastic phenotype in these tumours.

**Figure 2:**
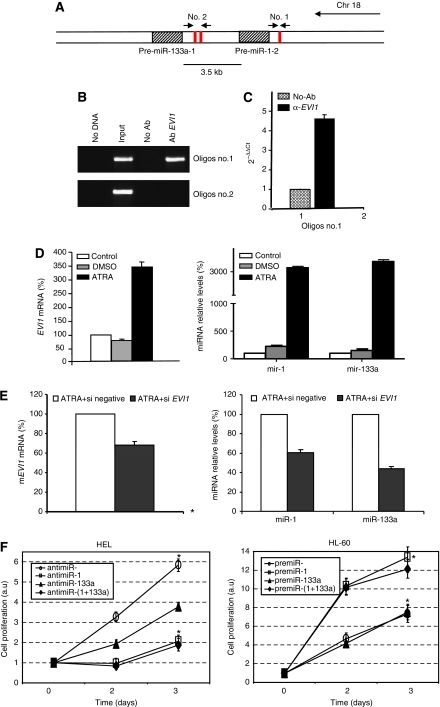
*EVI1* controls proliferation through miR-1-2 modulation. (**A**) Schematic representation of *EVI1*-binding sites and PCR design in the putative promoter of *miR-1-2* and *miR-133a-1*. (**B**) The PCR amplification of *EVI1*-immunoprecipitated DNA with the oligos no. 1 and oligos no. 2. Amplification of negative control (no DNA), input chromatin (input) and mock-immunoprecipitated chromatin (no Ab) were carried out as controls. (**C**) Quantitative real-time PCR was performed with oligos no. 1. Input chromatin was used as a normaliser. Error bars represent standard deviation. Results from a representative experiment, out of three, are shown. (**D**) Expression levels of mRNA *EVI1*, *miR-1-2* and *miR-133a-1* in cells treated with DMSO 1% or ATRA 1 *μ*M relative to P19 control cells, *β*-actin and snRNA U6B levels were used as normalisers for mRNA and miRNAs levels, respectively. (**E**) Quantification of mRNA *EVI1*, *miR-1-2,* and *miR-133a-1* in P19 transfected with a scramble or an *EVI1* siRNA, during the ATRA treatment. (**F**) HEL and HL-60 cells were respectively transfected with premiRs or anti-miRs, as indicated, and cell proliferation was measured at different time points. Untreated cells day 0 was given an arbitrary value of 1 and all the values were normalised with this group. Abbreviation: a.u.=arbitrary unit.

*miR-1-2* and *miR-133a-1* are clustered together in the same loci at chromosome 18 (Liu *et al*, 2007), suggesting that their transcription might be regulated by similar mechanisms. To elucidate whether *EVI1* acts as a transcription factor for these two microRNAs, we examine their putative promoters using bioinformatics prediction tools. Interestingly, there were several binding sites for *EVI1* in the upstream regions of both *miR-1-2* and *miR-133a-1* (Figure 2A). ChIP assays showed that *EVI1* was bound only to the region upstream *miR-1-2*, but not upstream *miR-133a-1* (Figure 2B). The occupancy of this site by *EVI1* was also confirmed by qRT–PCR (Figure 2C).

The P19 cell line expresses *EVI1* during ATRA-induced neuroectodermal differentiation, but not during DMSO-induced mesodermal. Thus, we assess whether *EVI1* induction in this model could also lead to *miR-1-2/133a-1* expression. Consistently, we could detect an increase in both *EVI1* and *miR-1-2/133a-1* levels during ATRA-induced differentiation (Figure 2D). Importantly, reducing the levels of *EVI1* by siRNA resulted concomitant decrease of these miRNAs (Figure 2E). Altogether, these data suggest a positive regulation of *miR-1-2* and *miR-133a-1* expression by *EVI1*.

Finally, we wanted to elucidate whether modulation of expression levels of these two miRNAs would have a functional outcome in AML cell lines. As *EVI1* overexpression has been linked with abnormal proliferation (Yuasa *et al*, 2005; Goyama *et al*, 2008) first, we assessed this function. We found that inhibition of miR-1-2 in the *EVI1*-positive HEL cell line induced a significant decreased in the proliferation potential of these cells. Interestingly, inhibition of miR-133a-1 resulted in a moderately decreased in the proliferation. When we transfected both of them together, we observed an effect that was very similar to the one produced by the antimiR-1 alone (Figure 2F). Of importance transfer of premiR-1, in the *EVI1*-negative cell line HL-60 resulted in a significant increased in the proliferation. However, transfer of premiR-133a-1 did not show any effect (Figure 2F). These data indicated that miR-1 could be a critical regulator of proliferation in *EVI1* overexpressing cell lines.

## Discussion

MicroRNAs have important roles in hematopoiesis, and also in the molecular pathogenesis of AML (Fabbri *et al*, 2008). To date, different miRNA signatures have been associated with defined AML cytogenetic subgroups. However, even though there is one study about miRNAs expression in relation with *EVI1* overexpresssion in mice (Dickstein *et al*, 2010), there are no studies analysing the differential miRNAs expression in AML patients with *EVI1* aberrant expression. Thus, this is the first study describing a miRNA profile associated with *EVI1* overexpression in human AML. This *EVI1*-associated signature consists of six different miRNAs, of which *miR-1* and *miR-133a-1* showed the highest correlation with *EVI1* levels in both cell lines and patient samples. Interestingly, Marcucci *et al* (2010) reported the upregulation of these two miRNAs in a set of AML patient with normal karyotype and a mutation in IDH2, indicating a possible role for these miRNAs in the malignant phenotype of AML patients. We found that *miR-1-2* expression was involved in proliferation but not *miR-133a-1*. Nevertheless, we do not discard the hypothesis that *miR-133a-1* could be involved in other functions such as inhibition of differentiation.

In conclusion, this study provides new insight in our understanding of the role of *EVI1* in AML. Our results demonstrate for the first time a functional relationship between *EVI1* and miRNAs expression. Moreover, uncovers a new pathway that could lead to the development of novel therapeutic approaches to treat *EVI1*-overexpressing leukaemia patients.
