# Supplementary material for: EVI1 controls proliferation in acute myeloid leukaemia through modulation of miR-1-2
Source: Br J Cancer. 2010 Sep 14;103(8):1292–6. doi: 10.1038/sj.bjc.6605874 (PMC2967053; doi:10.1038/sj.bjc.6605874)
Supplement: Supplementary Table 1 [file 6605874x1.doc]

**Table S1. Characteristics of 44 patients with AML**

**Characteristic Value No. of cases EVI1 overexpression (%)**

Age: median (range) 53 (2-80)

Sex: male/female 22/22 12 (54.5)/8 (36.6)

FAB classification

M0 3 3 (100)

M1 2 1 (50)

M2 10 3 (30)

M4 6 1 (16.6)

M5 8 5 (62.5)

M6 5 3 (60)

M7 1 1 (100)

NOS 9 3 (33.3)

Cytogenetics

Favorable group (n=2)

t(8;21) 2

Intermediate group (n=16)

Normal 10 3 (30)

Other 6 4 (66.6)

Unfavorable group (n=26)

Complex 7 2 (28.5)

3q 12 8 (66.6)

-7 7 3 (100)
